# Genomic variation in the vomeronasal receptor gene repertoires of inbred mice

**DOI:** 10.1186/1471-2164-13-415

**Published:** 2012-08-21

**Authors:** Elizabeth H Wynn, Gabriela Sánchez-Andrade, Keren J Carss, Darren W Logan

**Affiliations:** 1 Wellcome Trust Sanger Institute, Hinxton, Cambridge, UK

**Keywords:** Vomeronasal, Receptor, Olfaction, Pheromone, Behaviour, Genome sequencing, Single nucleotide polymorphism, Mouse

## Abstract

**Background:**

Vomeronasal receptors (VRs), expressed in sensory neurons of the vomeronasal organ, are thought to bind pheromones and mediate innate behaviours. The mouse reference genome has over 360 functional VRs arranged in highly homologous clusters, but the vast majority are of unknown function. Differences in these receptors within and between closely related species of mice are likely to underpin a range of behavioural responses. To investigate these differences, we interrogated the VR gene repertoire from 17 inbred strains of mice using massively parallel sequencing.

**Results:**

Approximately half of the 6222 VR genes that we investigated could be successfully resolved, and those that were unambiguously mapped resulted in an extremely accurate dataset. Collectively VRs have over twice the coding sequence variation of the genome average; but we identify striking non-random distribution of these variants within and between genes, clusters, clades and functional classes of VRs. We show that functional VR gene repertoires differ considerably between different *Mus* subspecies and species, suggesting these receptors may play a role in mediating behavioural adaptations. Finally, we provide evidence that widely-used, highly inbred laboratory-derived strains have a greatly reduced, but not entirely redundant capacity for differential pheromone-mediated behaviours.

**Conclusions:**

Together our results suggest that the unusually variable VR repertoires of mice have a significant role in encoding differences in olfactory-mediated responses and behaviours. Our dataset has expanded over nine fold the known number of mouse VR alleles, and will enable mechanistic analyses into the genetics of innate behavioural differences in mice.

## Background

The clone-based sequencing and assembly of the first high quality mouse genome, from the inbred C57BL/6J strain, revealed over 20,000 protein coding genes [1,2]. Approximately three quarters of these are direct 1:1 orthologues of genes identified in the human genome, but a number of gene families show striking rodent-specific expansions. These are enriched in genes associated with reproduction, reflecting an influence of sexual competition on the evolution of the mouse genome [1]. Mouse sexual behaviour and physiology is strongly regulated by pheromones: biochemical signals emitted by conspecifics that directly influence the behaviour or physiology of a receiving animal [3]. Accordingly, families of genes encoding protein pheromones are among those expanded in rodents [4-6]. This is mirrored by extensive species-specific expansions (with some selective losses and pseudogenisations) in gene families encoding rodent vomeronasal receptors (VRs) [1,7-12], which are putative receptors for pheromones [13,14].

The rodent VR repertoire consists of three distantly related families of G-protein coupled receptor, each expressed in sensory neurons of the vomeronasal organ in the nose: V1Rs, V2Rs and Formyl-peptide receptors (FPRs) [15-20]. Each vomeronasal sensory neuron (VSN) expresses a very restricted sub-set of VRs, typically one or a few of the same sub-family, thereby patterning each neuron to detect a limited number of ligands [15,19-23]. With a few exceptions [14,24,25], direct relationships between specific VRs and their pheromone ligands remain unresolved. However some general trends have emerged: neurons expressing V1Rs appear tuned to detect small volatile chemicals [26,27], those expressing V2Rs have been shown to detect proteinaceous ligands [13,28,29], while those expressing FPRs are stimulated by disease and inflammation-related peptides [19,20]. In addition, a significant subset VSNs detect chemical signals emitted by sympatric and predatory species, suggesting some VRs may be specifically tuned to allo-specific, instead of conspecific, cues [30-32].

The number of VRs identified in the C57BL/6J genome steadily increased with each draft genome assembly release, but has stabilised as the genome nears completion [10,11]. Recent analyses find 239 functional V1R genes [10], 121 functional V2R genes [11] and 7 FPR genes (of which 5 are expressed in the VNO [19,20]) distributed across the mouse genome in tightly clustered arrays. When their sequences are compared, the VRs within each cluster tend to fall within phylogenetically related clades, indicating that they expanded by localised duplication events [8,9]. Comparisons of VR clades across a range of mammals show that each species has a “semi-private” repertoire, consistent with a functional role as receptors for species-specific signals such as pheromones [7-11,23].

Although the variation in VR repertoire between divergent species is well documented [10], much less is known about the microevolution of VR repertoires between very closely related species. An analysis of 18 V1Rs between *M. m. musculus* and *M. spretus* revealed dynamic modulation in evolutionary pressures, including examples of positive selection and lineage-specific pseudogenisation [33]. Similarly, a recent study of 44 V1Rs in *M. m. musculus* and *M. m. domesticus* found evidence of genes shaped by negative selection and random drift, with a small proportion having evidence of positive selection [34]. Thus different rodent VR genes may be evolving under very different selective pressures, perhaps depending on the functional nature of their ligands; though whether these correlate within VR families, clades or even clusters is unknown.

Our understanding of intra-specific VR variation is even more limited. On one hand VRs may be expected to be under stabilising selection within a species, favouring low variation in receptor characteristics to ensure essential chemo signals (predator cues or sex-specific signals, for example [13,30,31]) are accurately detected and transduced. On the other hand, sexual selection driven by individual recognition may favour variation between individuals in both chemo signals and receptors [35]. Genome wide studies of copy number variation have reported significant variation in the size of VR clusters within inbred laboratory strains of mice [36,37], though accurate quantification of VR content and sequence level analyses of specific receptors are lacking. However, the value of using highly inbred, domesticated lab mice to study pheromone signalling has also been questioned [38]. Pheromone signalling may be largely redundant in laboratory mice releasing VR genes from selective constraint; alternatively these strains may offer a snapshot of the VR variation within the founder population, frozen by generations of inbreeding [36]. It is also possible that the very process of laboratory domestication has artificially shaped pheromone receptor content or variation, as was recently demonstrated in *C. elegans*[39].

The development of massively parallel sequencing (MPS) now offers the opportunity to rapidly and affordably resequence genomes from multiple individuals of the same species [40]. Recently the Mouse Genomes Project produced high coverage genome sequence of 17 inbred strains of mouse, including several laboratory-derived strains and wild-derived *M. musculus musculus, M. musculus domesticus* and *M. musculus castaneus* strains [41-43]. These are widely considered to be three subspecies of *Mus musculus*. *M. m. musculus* ranges across eastern Europe and northern Asia, *domesticus* is found throughout western Europe, the Middle East and north Africa, whereas *castaneus* extends throughout south and east Asia (reviewed in [44]). The laboratory-derived mouse strains are overwhelmingly *domesticus* in origin, with minor genomic contributions from *castaneus*x(~5%) and *musculus* (0.3%) [45]. The Mouse Genomes Project also sequenced the genome of a wild-derived *Mus spretus* strain [41-43]. *M. spretus* is a distinct species of short-tailed mouse with a sympatric range to *domesticus*, and serves as a genetic outgroup to the *Mus musculus* subspecies.

Here we interrogate the full complement of coding VR gene sequences in these 17 strains, compared to the C57BL/6J reference. We show that MPS can accurately resolve over half of mouse VRs, but that copy number variation (CNV) and non-specific short read mapping confounds complete repertoire analysis. We identify divergent patterns in the number and distribution of single-nucleotide polymorphisms (SNPs) within genes, clades and clusters of VRs, as well as between strains, and present evidence that the functional vomeronasal repertoire may vary significantly between *Mus* species and subspecies.

## Results

### Generating an accurate dataset of VR SNP variation

We collected SNP data in the open reading frames of 366 genes across 17 strains (a total of 6222 genes and over 9 Mb of DNA sequence), each encoding a potentially functional VR identified in the C57BL/6J reference genome. We then adopted a conservative parsing strategy to ensure accurate and statistically robust data, removing genes on the basis of ambiguous read calls, read mapping quality and incomplete gene coverage (see Materials and Methods and Additional file 1: Table S1 for detail). In total 2856 VR genes (45.9%) were excluded leaving a dataset of 3366 full-length VRs: 1980 V1Rs, 1301 V2Rs and 85 FPRs. Within the coding region of these genes we identified 11,207 SNPs that differ from the C57BL/6J reference sequence (Table1, Additional file 2: Table S2).

**Table 1:** Variation in vomeronasal receptor genes identified by massively parallel sequencing

		**DNA sequence**			**SNPs**						
**Strain**	**VRs after parsing**	**Resolved sequence (in Kb)**	**Unresolved sequence %**		**All SNPs**	**Non-synonymous SNPs (%)**		**Private SNPs (%)**		**Truncating**	
C57BL/6NJ	203	316.936	215.204	(40.4)	1	1	(100)	1	(100)	0	(0.00)
129S1/SvImJ	200	314.377	217.763	(40.9)	418	238	(56.9)	3	(0.72)	2	(0.84)
129S5SvEvBrd	201	312.532	219.608	(41.3)	396	224	(56.6)	1	(0.25)	2	(0.89)
129P2/Ola	202	315.127	217.013	(40.8)	416	231	(55.5)	24	(5.77)	4	(1.73)
A/J	200	307.621	224.519	(42.2)	318	191	(60.1)	36	(11.3)	4	(2.09)
AKR/J	200	309.55	222.59	(41.8)	311	170	(54.7)	7	(2.25)	4	(2.35)
BALB/cJ	202	315.94	216.2	(40.6)	301	184	(61.1)	1	(0.33)	4	(2.17)
C3H/HeJ	202	314.41	217.73	(40.9)	216	122	(56.5)	0	(0.00)	2	(1.64)
CBA/J	203	316.936	215.204	(40.4)	249	144	(57.8)	3	(1.20)	2	(1.39)
DBA/2J	201	311.824	220.316	(41.4)	370	216	(58.4)	22	(5.95)	2	(0.93)
LP/J	202	314.371	217.769	(40.9)	521	289	(55.5)	11	(2.11)	3	(1.04)
NOD/ShiLtJ	200	314.167	217.973	(41.0)	333	187	(56.2)	11	(3.30)	2	(1.07)
NZO/HILtJ	199	313.186	218.954	(41.2)	565	333	(58.9)	36	(6.37)	7	(2.10)
**Lab-derived**	2615	4076.977	2840.843	(41.1)	4415	2530	(57.3)	156	(3.53)	38	(1.50)
PWK/PhJ	194	298.987	233.153	(43.8)	1409	789	(56.0)	508	(36.0)	6	(0.76)
CAST/EiJ	183	280.912	251.228	(47.2)	1402	808	(57.6)	571	(40.7)	8	(0.99)
WSB/EiJ	202	314.389	217.751	(40.9)	462	291	(63.0)	84	(18.2)	4	(1.37)
SPRET/EiJ	172	265.669	266.471	(50.1)	3519	1976	(56.2)	2619	(74.4)	20	(1.01)
**Wild-derived**	751	1159.957	968.603	(45.5)	6792	3864	(56.9)	3782	(55.7)	38	(0.98)
**Total**	3,366	5,236.93	3,809.45	(42.1)	11,207	6,394	(57.1)	3,938	(35.2)	76	(1.19)

To assess the accuracy of our dataset, we compared VR sequences identified by MPS with those generated by traditional long-read, capillary sequencing. Recently Kurzweil *et al.* reported the sequencing of a cluster of V1R genes and pseudogenes from a SPRET/EiJ BAC library [33]. We identified nine BAC sequenced V1R genes (8.1 kb of sequence) that was also in our parsed dataset. Assuming no error in the BAC sequencing, we calculate a false positive SNP call rate of 1.3%, with a false negative rate of 0.5%. This equates to 99.95% accuracy in base-pair calling. We were also able to identify four genes (3.6 kb of sequence) that we had excluded for failing to meet our quality threshold. In these genes the false-positive and false-negative SNP rates are significantly higher, 5% and 17.5% respectively, which supports the use of our parsing strategy to generate an accurate, albeit conservative, SNP catalogue for this study.

### VR SNP distribution among mouse strains

**Figure 1:**
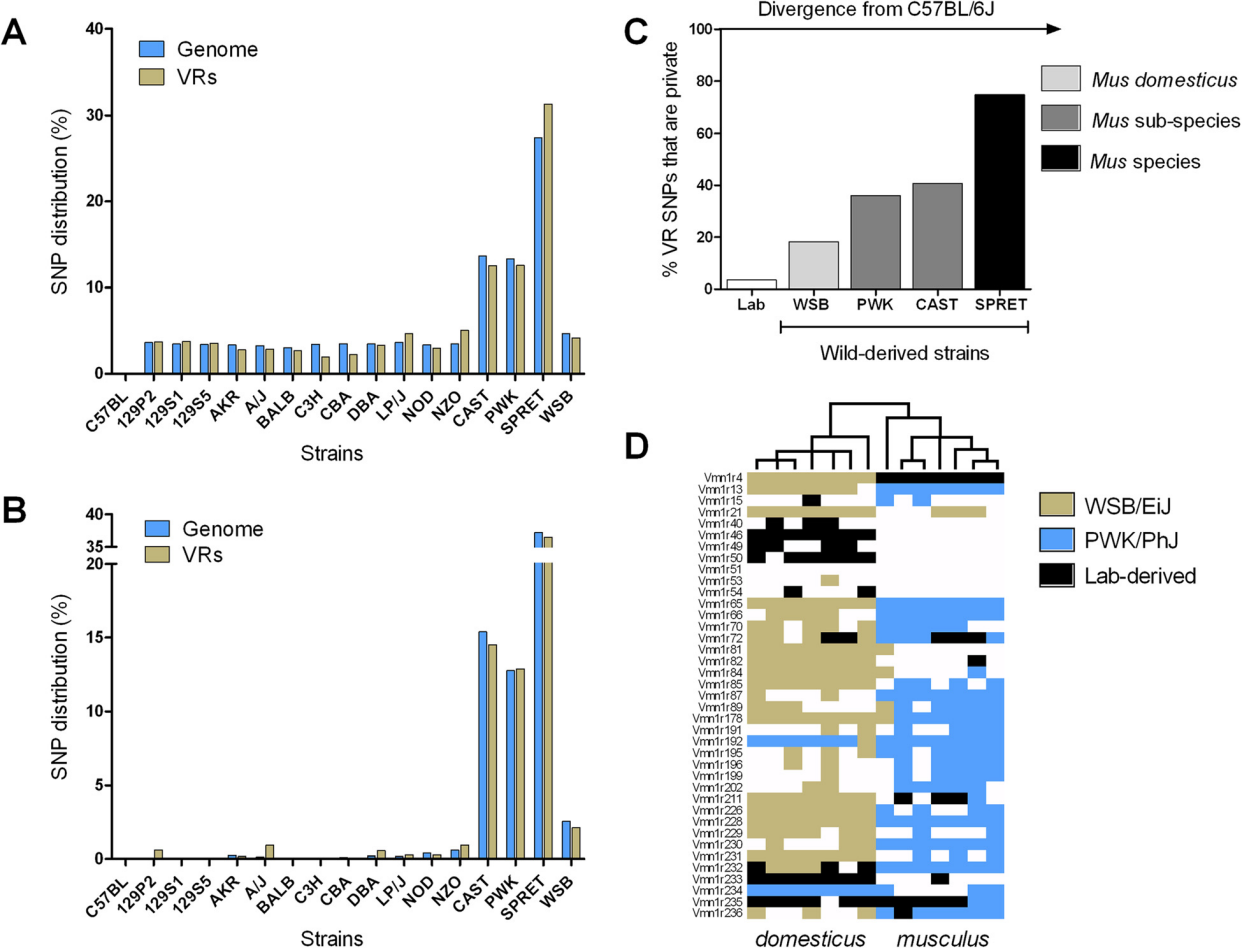
**The distribution of SNPs within VRs between inbred strains of mice.** (**A**) The distribution of the total number of SNPs identified within the coding region of vomeronasal receptor genes (VRs) compared with the total number of SNPs in the entire accessible genomes. (**B**) A similar comparison showing the relative distribution of private SNPs (those that are unique to one strain). The distributions of SNPs across entire accessible genomes were calculated from data in [42]. (**C**) The relative proportions of the VR SNPs in each strain that is private. The data from the 13 laboratory-derived strains (Lab) are pooled. Increasing evolutionary distances from the C57BL/6J, mainly *domesticus*-derived, reference strain are indicated with increasingly darker shading. (**D**) A heat map showing whether WSB/EiJ, PWK/PhJ or another laboratory-derived strain matches V1R sequences from 14 wild-caught isolates described in [34]. The V1R genes are arranged vertically in order and the isolates are arranged by hierarchical clustering (indicated top), resulting in two clusters consistent with the subspecific origin of the isolates.

The 17 sequenced strains were carefully selected to ensure maximum utility among the research community, and include 14 common lab strains, as well as strains more recently derived from wild-caught mice of other *Mus* species and subspecies [40,42,43]. We first determined whether the distribution of SNPs in VRs showed unusual distribution patterns. As expected, wild-derived strains harbour a greater number of VR SNPs than classical laboratory strains, each in proportions similar to the rest of the genome (Figure1A) [42]. The *musculus* (PWK/PhJ) and *castaneus* (CAST/EiJ) wild-derived strains (both are subspecies of *M. musculus*) have approximately 4 times more SNPs in VRs. The *spretus* strain (SPRET/EiJ), an entirely distinct *Mus* species, has approximately 10 times more (Figure1A, Table1). However, the total number of SNPs overestimates the diversity of unique VR alleles within the strains of mice as many variants are likely to be shared, particularly between laboratory-derived strains. We therefore determined the repertoire of private VR SNPs within each strain (Figure1B, Table1). Again, the distribution of private SNPs closely mirrors that reported across the whole genome [42]. There are very few private SNPs in VRs from lab-derived strains, but a large proportion of the VR SNPs in wild-derived strains are private; the number increasing with greater evolutionary distance from largely *domesticus*-derived reference strain (Figure1C). Strikingly, WSB/EiJ, the *Mus domesticus* wild-derived strain [46], has a similar number of total VR SNPs to the lab strains but a greater proportion of these (18.2%) are private (Figure1A, C, Table1). This difference illustrates the restricted genomic diversity in the VRs of classical laboratory-derived strains compared to wild-derived mice, and mirrors a similar restriction in diversity of VR ligand expression [38]. Together these may impact on the suitability of laboratory strains for studying the neural mechanisms underpinning social recognition, but do wild-derived strains better approximate the VR repertoire of wild individuals? To test this we utilised a recently published dataset of 44 V1R gene sequences, each from 7 *M. m. musculus,* and 7 *M. m. domesticus* wild isolates [34], and asked whether each wild mouse has a VR haplotype that matches the *musculus* (PWK/PhJ) and *domesticus* (WSB/EiJ) wild-derived strains (Figure1D). We found that on average half of the isolates had at least one allele from its respective wild-derived strain (measured across 39 genes). We identified only one VR gene, *Vmn1r51* (*V1ra1*), in which no wild isolate matched the sequence from any of the 17 inbred strains in this study.

### Functional variation in VRs

**Figure 2:**
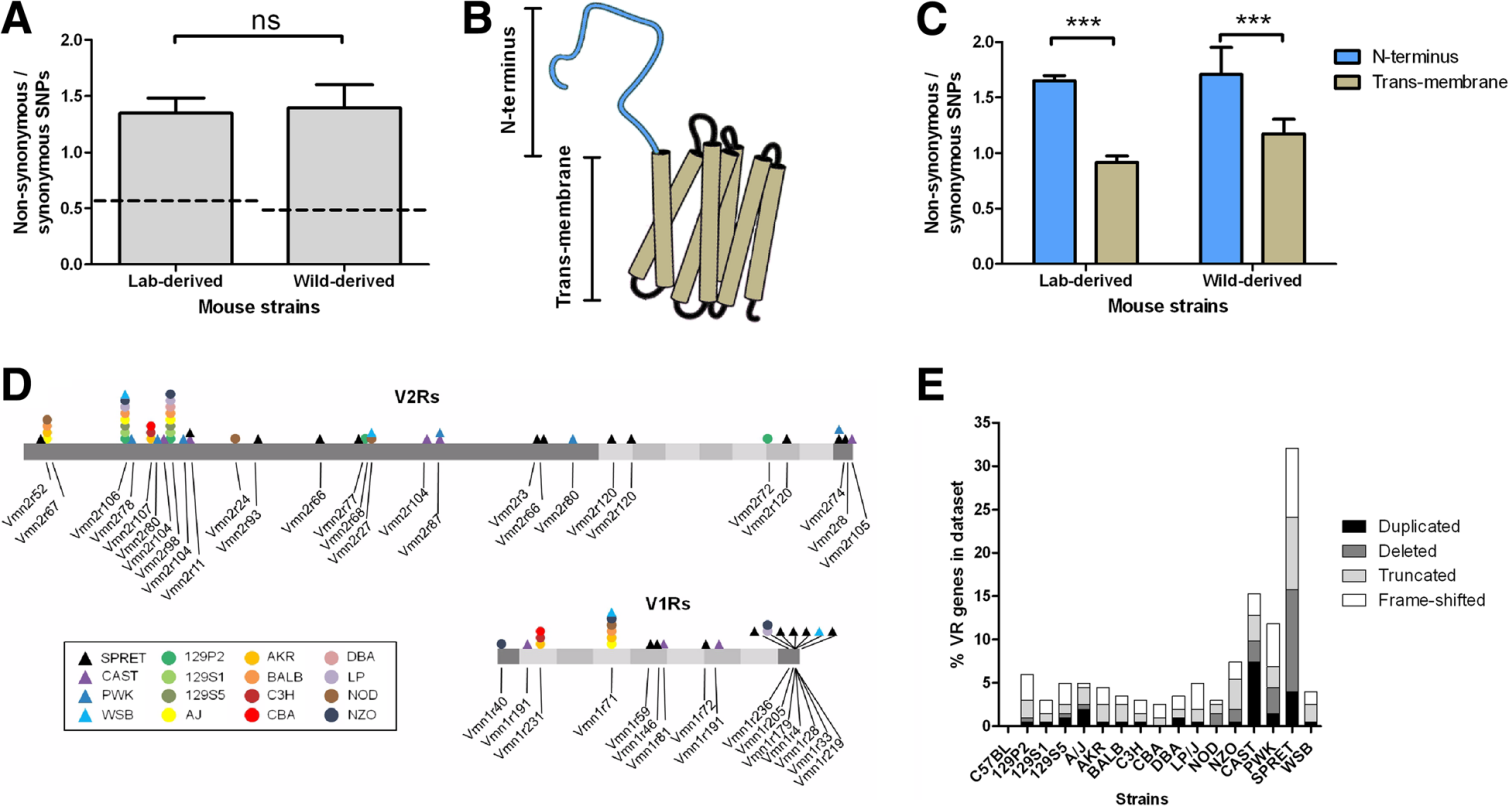
**Evidence of dynamic selective pressures on VR repertoires in both laboratory- and wild-derived strains.** (**A**) The ratio of non-synonymous to synonymous SNPs in VRs is similar between wild- and lab-derived strains (mean + SEM. Two-tailed t-test, P = 0.622), but higher than the genome averages (dashed lines: lab-derived = 0.54, wild-derived = 0.49 calculated from the data in [42]). (**B**) A schematic representation of the proposed secondary structure of a V2R receptor protein. The N-terminal domain (blue) is likely to be extracellular allowing it to interact with its pheromone ligand. (**C**) The normalised distribution of SNPs reveals significantly more functional variation is found in the N-terminal domains of V2Rs than trans-membrane domains (Two-way ANOVA, variance in domain F_1,14_ = 138, P < 0.0001, variance in strain F_1,14_ = 1.5, P = 0.24. Bonferroni *post hoc* test, *** = P < 0.001). (**D**) The distribution of termination codons across VRs. The VR domains are indicated by shading (N- and C-termini in dark grey, trans-membrane domains in lighter greys). The relative positions of the termination codon found in each gene are indicated beneath. The strain each termination codon is found in is indicated by colour and shape (lab-derived strains by circles, wild-derived strained by triangles), above. Note some termination codons are found in multiple strains and some genes have multiple termination codons (e.g. *Vmn2r120*). (E) The proportion of genes analysed with evidence of truncation, frame shift, deletion or duplication, suggesting the functional VR repertoire is highly variable between strains of mice. Any gene that contained more than one of these (for example, was truncated and had a deletion) was counted only once in the following order: duplication, deletion, truncation, frame shift.

SNPs within the coding region of genes can be divided into two classes based on functional consequence: those that are predicted to alter the resultant amino acid sequence (non-synonymous substitutions) and those that do not influence the sequence of the protein product (synonymous mutations). We identified 6394 non-synonymous SNPs, 57% of the total, and found no difference in the ratio of non-synonymous to synonymous SNPs between wild-derived and lab strains (Figure2A). However the density of non-synonymous SNPs in VRs is relatively high: on average one was found every 273 codons of VR gene, which is 2.3 times more frequent than when calculated across the whole coding genome (one every 634 codons). This relative enrichment is specific to non-synonymous SNPs, as the frequency of synonymous SNPs in VRs is similar to that of the genome average (one synonymous SNP every 363 codons of VR compared to one every 325 codons, on average, across the genomes). A large increase in non-synonymous SNPs could indicate that VR genes have been completely released from selective constraint in domesticated strains, in which case non-synonymous SNPs would randomly accumulate throughout the length of VR genes; or that the restraint is weakened in which case some evidence of selection may remain apparent in regions of the gene. We therefore ascertained whether the distribution of variation is uniform within VR domains.

V1Rs and FPRs are members of the class A rhodopsin-like receptor superfamily [47]. They are encoded by small, typically single exon, genes and dominated by the core seventrans-membrane domains with only a small extracellular domain [48]. V2Rs belong to the class C glutamate-like receptor superfamily; they are multi-exonic and are characterised by a long N-terminal extracellular domain. This region is predicted to be involved in ligand recognition and, in contrast to the highly conserved seven trans-membrane domains, is highly variable between different members of the V2R family (Figure2B) [9,49]. We divided 1220 V2R genes from 16 strains into N-terminal and trans-membrane domains, and calculated the ratio of non-synonymous to synonymous SNPs for each. In both laboratory-derived and wild-derived strains, we found a statistically significant bias in functional variation towards the N-terminal domains (Figure2C). This is consistent with an enhanced selective constraint on the conserved seven trans-membrane domains and suggests that V2Rs, at least, are not entirely redundant in domesticated mice.

**Figure 3:**
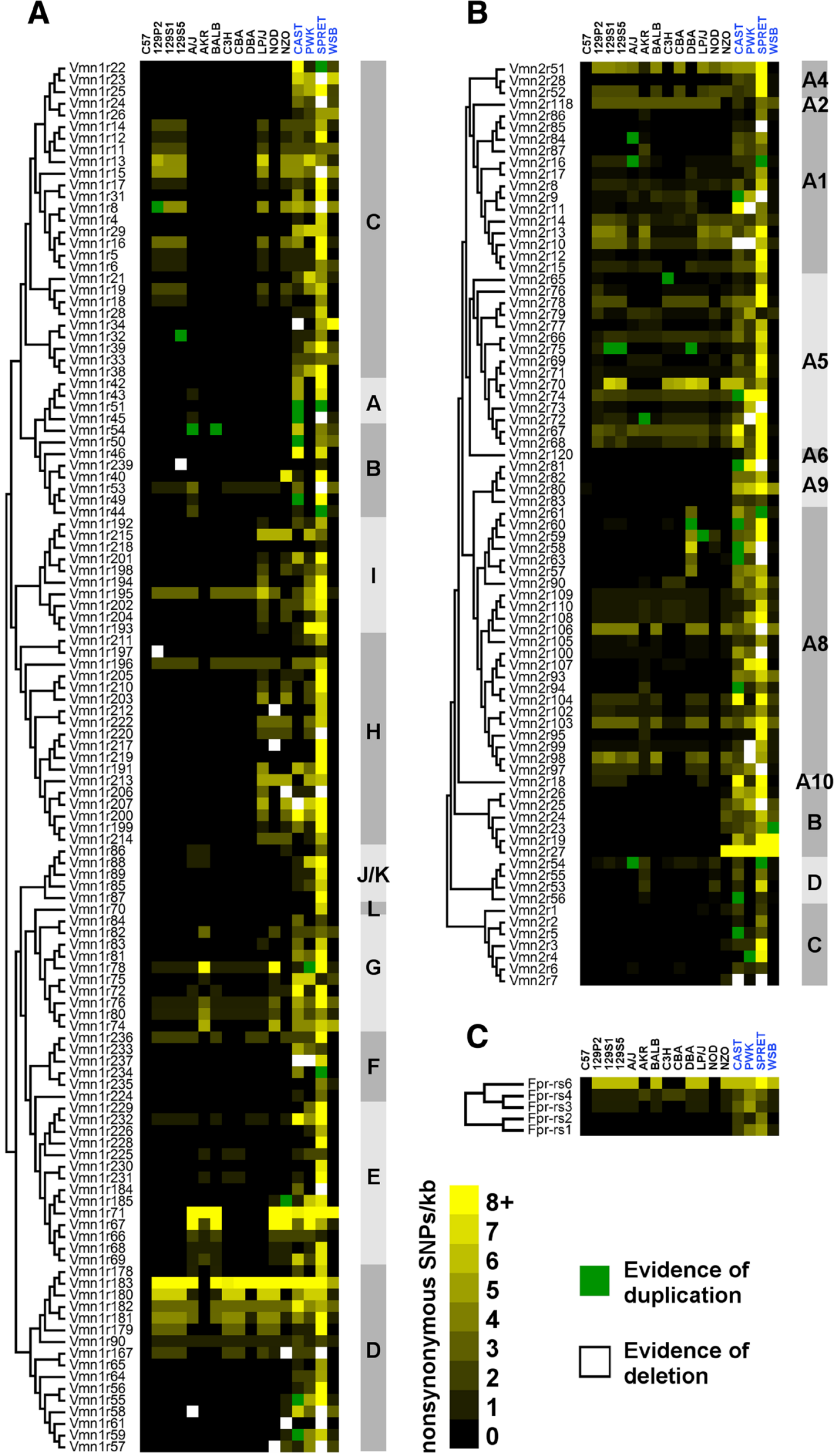
**A heat map of functional variation by vomeronasal receptor class and phylogeny.** The amount of functional variation in all genes in our parsed dataset is represented by colour (black to yellow). The values are normalised between and within strain, as the number of non-synonymous SNPs in a kilobase of coding sequence. Each row represents a gene from the (**A**) V1R (**B**) V2R and (**C**) FPR sub-families, arranged by phylogeny (left). Where receptor sub-families have been further divided into recognised phylogenetic clades, those are indicated (right) in alternating shade of grey. The clade nomenclature is as described [10,11,19,20]. Each column represents a strain, divided into lab-derived (left, black text) and wild-derived (right, blue text). Genes removed from the dataset because of evidence of duplication (green), or deletion (white), are also indicated.

To further assess the functional constraint on VRs, we next determined how many non-synonymous SNPs created a termination codon that results in a premature truncation of the reading frame, potentially rendering the mature receptor non-functional. We identified 76 new termination codons that collectively truncate 70 VR genes (Figure2D). Some truncating SNPs are shared, especially among laboratory strains; but when aggregated we found that 18.2% of the VR genes analysed are truncated in at least one strain. Shifts in reading frame, as a result of insertions or deletions of a few nucleotides (indels), are also likely to result in a non-functional protein. We that found that a further 14.3% of the genes analysed are frame-shifted in at least one strain (Figure2E). We were also able to identify evidence of larger (>100 bp), lineage-specific deletions in 44 VRs and lineage-specific duplications in 42 VRs (Figure2E, Figure3, Additional file 3: Figures S1 and Additional file 4: Figure S2). Taken together, it is clear that the functional repertoire of pheromone receptors varies significantly between inbred strains of mice and the C57BL/6J VR reference set.

### SNP distribution between and within VR families

**Figure 4:**
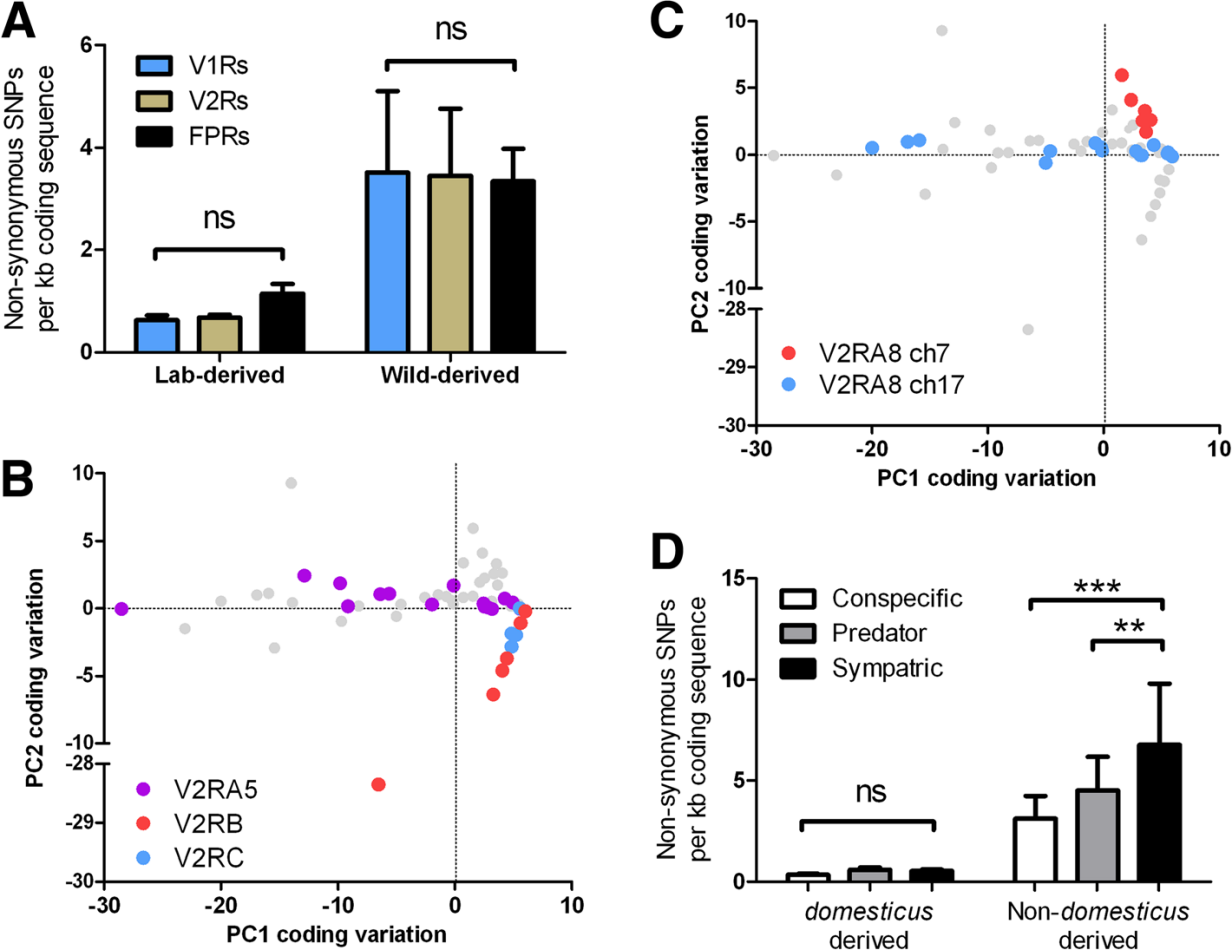
**Correlations between receptor coding variation, clade, chromosomal location and function.** (**A**) The accumulation of non-synonymous SNPs does not differ between VR sub-families within either lab-derived or wild-derived strains (Mean + SEM. Two-way ANOVA, variance in receptor subfamily F_2,28_ = 0.312, P = 0.734, variance in strain F_1,28_ = 17.01, P = 0.001. Bonferroni *post hoc* tests of all combinations within strains, ns = P > 0.05). (B,C) Non-synonymous SNP distribution within lab-derived strains displays correlations with V2R organisation byprincipal component (PC) analysis. The two most important PCs (accounting for 72% of the variation in the data) are plotted against each other for all V2R genes. Examples of V2R genes within select cladesare highlighted, having different directional clustering across the PC axes: (**B**) V2RB and V2RC (red and blue) differ from V2RA5 (purple). (**C**) Differences in grouping is also observed within a clade that has two arrays of receptors located on different chromosomes (V2RA8 on chromosome 7, red and on chromosome 17, blue). (**D**) V2Rs that detect sympatric cues in *domesticus* are more variable in other *Mus* species or subspecies, than those that detect predator or conspecific cues. Mainly *domesticus*-derived strains were pooled and those strains from another *Mus* species or subspecies were pooled. The functional classification of each V2R gene is as described [32] (Mean + SEM. Two-way ANOVA, variance in function F_2,28_ = 17.29, P < 0.0001, variance in strain F_1,28_ = 27.8, P = 0.0001. Bonferroni *post hoc* tests of all combinations of functional class within strainpools, ns = P > 0.05, ** = P < 0.01, *** = P < 0.001).

Cellular expression of the three known sub-families of vomeronasal receptors, V1R, V2R and FPRs is segregated within the VNO, but it is not yet known whether the behaviours they regulate are functionally distinct and therefore under different selective pressures. We therefore analysed the sequences of each sub-family independently to assess for differences in genomic variation, and considered only those SNPs with the potential to have a functional consequence. After normalisation for gene length, we find the mean abundance of non-synonymous SNPs across all strains to be very similar between V1Rs and V2Rs in both lab-derived strains (0.635/kb and 0.681/kb) and wild-derived strains (3.516/kb and 3.429/kb). The small family of FPRs have increased mean abundances in lab strains, but the increase is not significant and largely the consequence of one unusually variable outlier, *Fpr-rs6* (Two-way ANOVA, variance in receptor class F_2,28_ = 0.312, P = 0.734) (Figure3, Figure4A).

As we found no evidence of differences between entire VR sub-families we next tested whether there was statistical correlation in variation within sub-families, based on the hypothesis that closely related receptors are likely to be activated by closely related ligands that may, in turn, regulate similar types of behaviours. Considering wild-derived and lab-derived strains independently, principal component analyses (PCA, an unsupervised linear feature extraction method that discovers the directions of maximal variances in data) found highly significant correlations between non-synonymous SNP distribution and phylogeny within lab strain V2Rs (Two-way ANOVA, variance by V2R clade F_12,858_ = 17.99, P < 0.0001). For example, over 90% of the V2R variation in lab-derived strains can be represented by four principal components (eigenvalues <2). Grouping of VRs by cluster is apparent by the first two components in PCA, as shown by V2RB and V2RC, which are highly conserved among lab-derived strains, and V2RA5, which is highly variable (Figure3, Figure4B). Similar correlations can be observed within V1Rs and wild-derived strains (Two-way ANOVA, variance by V2R clade F_12,264_ = 5.52, P < 0.0001) and even within closely related receptor clades, such as the two non-contiguous VR clusters that together generate V2RA8, *Vmn2r57-61* on chromosome 7and *Vmn2r90-110* on chromosome 17 (Figure3, Figure4C). Physically clustered VRs may therefore act as “units” of variation, with constituent receptor genes sharing characteristics of SNP accumulation or resistance. This implies that at least some clusters of VRs were shaped by contrasting selective pressures before or during strain derivation, but can these differences be reconciled with what is currently known about VR function?

### SNP distribution between functional classes of V2R

Recently Isogai *et al*. undertook a comprehensive survey of immediate early gene upregulation in VSNs after exposure to olfactory stimuli derived from conspecifics and a diverse range of other animals. They then identified the VRs expressed in these VSNs and showed that individual V2Rs appear specifically tuned to detect the nature of the signal emitter, whereas V1Rs are activated by cues from multiple species [32]. Although both the specific chemosignals and the behavioural consequences of detection remain unresolved, these groups of V2Rs represent the first functional classification of VRs in mice. The receptors can be classed as those that detect conspecific odour cues (soiled bedding from the same species of mouse), predators (including birds of prey, snakes and mammals) and sympatric non-predatory rodents (including *M. spicilegus* and *M. m. musculus*) [32]. We reasoned that these functional classes of V2R would likely be under divergent selective pressures within and between species/subspecies of mouse. Indeed, within the strains that represent different species/subspecies, the V2Rs that detect sympatric cues differ significantly more from the C57BL/6J reference than those that detect either conspecific (Two-way ANOVA, Bonferroni *post hoc* test, P < 0.001) or predator cues (P < 0.01) (Figure4D). However, within *domesticus* strains there is no significant difference in variation by function (P > 0.05).

## Discussion

### Massively parallel sequencing of VRs

To our knowledge, this is the first application of MPS to specifically resequence and analyse vomeronasal receptor genes. Therefore we first assessed whether current technology was capable of accurately resolving the sequences of these highly homologous, clustered sequences. Across entire genomes, 77-87% of reference sequence can be resolved, depending on the strain [42]. In contrast we were able to resolve between 50-60% of known coding VR sequence with high accuracy. While some of the unresolved sequence is certainly a consequence of strain-specific VR deletions or duplications, the majority is likely due to the challenge of uniquely mapping short sequence reads to highly repetitive genes [50]. Two strands of evidence support this conclusion: firstly the VR clades with the highest sequence homology are enriched in the unresolved sequence, and these are also typically unresolved across all strains. Secondly in C57BL/6NJ, a sub-line of the C57BL/6J reference strain that has just one SNP difference over 300 kb of VR sequence, only 60% resolution was achieved. It is improbable that such a large proportion of the VR repertoire would differ between such closely related sub-strains while virtually no SNPs were accumulated. Instead it is likely that 60% resolution is a baseline value for mapping reads to VRs in this dataset. In comparison, we were able to resolve only 50% of the *spretus* VRs, which may indicate that as much as one tenth of the *domesticus* functional VR repertoire is missing in *spretus* (for an example, see Additional file 3: Figure S1). MPS is therefore currently only partially effective for accurately resequencing VRs in mouse. However, as mapping success is largely influenced by read length, the rapid development of MPS technology suggests that more of the VR repertoire will be resolved in future resequencing efforts [51].

### Considerations in using inbred strains for behavioural research

Domestication by hundreds of generations of inbreeding has resulted in strains of laboratory mouse that display constrained innate behavioural responses when compared to wild (or recently wild-derived) relatives [52-54]. We find that variation within VRs to be similarly restricted within lab-derived strains, reinforcing the genomic limitation of these strains in studies of olfactory-mediated mouse behaviour, especially those that involve individual recognition or the detection of genetic relatedness by olfaction [35,38,55]. Nevertheless, inbred lab-derived strains do differ from one another in some olfactory and/or pheromone mediated behaviours [14,56,57]. Strain-specific variance in genetically-encoded pheromone production will certainly account for some of these differences [5,14,28,58], but we also find evidence of unusual strain-specific differences in VRs that may influence behaviour. For example, in some cases unusual clustering of rare privates SNP scan be observed. 129P2/OlaHsd has no private SNPs in V1Rs but 8% of the V2R SNPs in that strain are private and almost all are clustered within a single VR clade, V2RA5. Furthermore 129P2/OlaHsd, which serves as the background strain for many knockout mice lines [40], also has over 5% of the C57BL/6J functional VR repertoire either truncated, frame-shifted or deleted. It is important to stress that the truncating SNPs and frame-shifting indels described here do not definitively denote pseudogenisation, as alternative splicing or termination codons near the 3′ end of genes may still result in fully functional receptors being produced. Nor can we confidently report this the upper limit of VR pseudogenisation, as other SNPs that result in non-conservative amino-acid substitutions may also result in functionally disabled genes and are not recorded here. Nevertheless, these data will assist strain selection and control when conducting or comparing experiments on olfactory-mediated behaviour in lab mice. The VR variation reported here may also explain conflicting reports in the literature about the effectiveness of some putative pheromones [59].

### Selection on VRs in inbred mice

We found that the distribution of SNPs in lab-derived strains is not random, but correlated with VR clade and genomic clusters, suggesting at least some VRs remain under selective constraint during the process of domestication by humans. It is tempting to speculate that those clades with the least variation mediate critical pheromone-mediated behaviours, such as reproduction or maternal care, which are essential even in a laboratory environment. However the paucity of VRs with defined ligands or functions makes it impossible to draw firm conclusions at this time. Of the VRs with known ligands [14,20,24,25], *Vmn2r116* (also known as *V2rp5*) was not resolved in this study. Two others, *Vmn2r26* (also known as *V2r1b*) and *Vmn1r49* (also known as *V1rb2*), are highly conserved among lab-derived strains. The FPRs display a range of variation, but it is presently unclear what behaviours these receptors mediate. It is apparent, however, that the receptors in the atypical V2RC clade, which may act as functional co-receptors with other V2Rs [60], are among the most conserved VRs.

As a corollary to selection for essential VRs, receptors that are widely inactivated may be either disadvantageous (or inconsequential) to domesticated environments. Recently a study demonstrated examples of acquired pheromone resistance during domestication of *C. elegans*, by deletion of dauer pheromone receptor genes [39]. Similar pressures may have acted on VRs during mouse domestication, especially as a number of pheromone-mediated behaviours, such as aggression or inbreeding avoidance, are likely to be disadvantageous in a laboratory environment. Unfortunately, because the reference genome is also from a laboratory-derived inbred strain, this study is not well suited to detect such deletions. However mapping the VR sequences described here to a *de novo* genome assembly of a wild-derived *domesticus* mouse genome would permit the identification of novel VRs missing in domesticated mice.

### VRs between species and subspecies of mice

We used four wild-derived strains as representatives of “wild” species and subspecies of *Mus*: *M. musculus musculus, M. musculus domesticusM. musculus castaneus* and *M. spretus*. These strains are also inbred to homozygosity, albeit through fewer generations, and therefore under-represent the true genomic variation in wild-caught mice. Nevertheless here we have shown that they faithfully capture many of the pheromone receptor gene sequences found in wild individuals [34], supporting their use as genetically controlled, “pseudo-wild” animals to investigate VR function. However, some wild-derived inbred strains have genomic contamination with classical strains [45] and our data reveals specific examples of this when compared with sub-sets of VRs from wild-caught mice [34]. Most strikingly, the entire V1RA and V1RB receptor cluster in the *musculus* wild-derived strain (PWK/PhJ) are identical to the C57BL/6J reference (see Figure3A) and do not match any of the alleles from wild *musculus* individuals (see Figure 1D). Even with such cases of introgression between subspecies, we were able to identify significant differences in VR variation depending on the nature of their ligand. Most species/subspecies of *Mus* will encounter olfactory signals from similar predatory species that activate subsets of VRs resulting in defensive behaviours [30-32]. There is relatively little coding variation in those receptors between *domesticus* and non-*domesticus* derived strains, consistent with strong selective pressure. In contrast, some VRs are tuned to detect cues from other sympatric mouse subspecies [30,32]. The functions of these receptors are unknown, but they may mediate a behavioural barrier that contributes to reproductive isolation between subspecies [61]. We found that, collectively, these receptors are more variable in non-*domesticus* derived strains than those detecting predators or conspecifics. *Vmn1r67* (also known as *V1re10*) has been proposed as a candidate for mediating subspecific recognition between these subspecies [62]. The gene was not included in the analysis described above, but we did identify the same 24 SNP differences between *musculus* and *domesticus* alleles reported previously. Unexpectedly, four laboratory-derived strains (A/J, BALB/cJ, NOD/ShiLtJ and NZO/HlLtJ) also share the presumably introgressed *musculus* allele while the others have the *domesticus* allele (with one additional SNP). Thus it may now be possible to exploit these introgressions to delineate a function for this unusually variable receptor in subspecific recognition, using more commonly available laboratory-derived mice.

## Conclusions

Here we have resolved, analysed and compared the full coding sequences of over 3300 pheromone receptors of unknown function, from 17 strains of inbred mice using massively parallel sequencing data of whole genomes. We describe complex patterns of non-random sequence variation that indicate these receptors are under divergent selective pressures that correlate with proposed ligand, phylogeny, chromosomal clustering, and protein domain. Moreover, we demonstrate that VR protein sequences are unusually variable between mice, and that the repertoire of functional genes differs significantly, mirroring the inter-individual diversity in pheromone ligands. Together this supports a two-component model in which differences in both receptor and pheromone ligand sequence (and expression) may together genetically encode a diverse range of innate responses. We anticipate, as VRs are increasingly deorphanised in mice, the data described here will be invaluable for investigation into the genetic basis of behavioural differences.

## Methods

### Data collection

We retrieved VR cDNA sequences from 239 V1R [10], 121 V2R [11], and 7 FPR sequences [19,20], and use the nomenclature of those authors. We used BLAT to determine the genomic co-ordinates of each gene exon in the NCBIM37 mouse reference genome. One gene, *Vmn2r119*, was excluded because it mapped to the same location as *Vmn2r118*. We then used Mouse Genomes Project SNP query tool to compare the genome sequences of 17 strains (European Nucleotide Archive: [ENA: ERP000034–ERP000050]) across each exon of each VR gene, relative to the NCBIM37 reference [63]. Full detail of how each strain was sequenced and mapped, and how the SNPs were called is described by Keane *et al.*[42]. Briefly, frame shifts between 1 and 50 bp were called by Dindel [64]. SNPs were called by agreement of two or more of four different SNP calling algorithms: Samtools varFilter 8, Genome Analysis Toolkit 10, iMR and QCALL 11. The SNP query tool parsed each SNP with a quality score >10, and then calculated the predicted transcriptional consequence from its location in an open reading frame: reporting synonymous, non-synonymous or ambiguous SNPs, and additionally when a termination codon is gained or lost. We then used Look-seq with a MAPQ cutoff >30 to identify and exclude genes lacking sequence coverage across the entire coding region [65]. These included clusters of genes with extremely high homology (e.g. entire V1RD and V2RA4 clades), and genes that appear to have specific deletions in only some lineages (e.g. see Additional file 3: Figure S1). Only the latter are described as deletions in Figures 2E and 3. Genes that had > 50% ambiguous SNP calls were also excluded. Although these ambiguous calls are experimentally unresolved, they are typically the result of multiple haplotypes in the sequenced strain mapping to the same location in the reference strain (e.g. see Additional file 4: Figure S2), which is consistent with a duplication of that locus. These are described as duplications in Figures 2E and 3. Finally, if an orthologous gene was excluded in more than three strains for any of the above reasons, SNPs from that gene were then excluded from all other strains for statistical purposes. Additional file 1: Table S1 shows the number of genes excluded for each reason. All the SNPs in the dataset used in this study are listed in Additional files 5,6 and 7. The mouse strains used in this study are: 129P2/OlaHsd, 129S1/SvImJ, 129S5SvEvBrd, A/J, AKR/J, BALB/cJ, C3H/HeJ, C57BL/6NJ, CAST/EiJ, CBA/J, DBA/2J, LP/J, NOD/ShiLtJ, NZO/HlLtJ, PWK/PhJ, SPRET/EiJ and WSB/EiJ.

### Heat-mapping

SNP density heat maps were generated using Cluster and Java Treeview [66,67], and arranged using the phylogeny reconstructed using MEGA from aligned C57BL/6J VR cDNA sequences using the neighbour-joining method with the Kimura-2 parameter model of substitution [68]. The heat-mapping of wild-caught isolates was arranged by hierarchical clustering with complete linkage.

### VR domains

TMHMM was used to predict the location of the beginning of the first trans-membrane domain in each V2R [69]. The number of synonymous and non-synonymous SNPs either side of this location was calculated and normalised to the domain size. SNPs located in the short loops between each trans-membrane span were included in the seven trans-membrane domain count. The relative location of truncation codons were also identified using TMHMM.

### Comparisons with published sequence data

We aligned the V1R sequences reported by Kurzweil *et al.*[33] to a *de novo* scaffold of the SPRET/EiJ genome [41] using CLUSTAL W2 [70] and identified each gene by sequence identity and synteny. The nine gene pairs compared were YUA. 5 (*Vmn1r40*), YUA.3 (*Vmn1r42*), YUB.2 (*Vmn1r43*), YUA.4 (*Vmn1r44*), YUB.1ps (*Vmn1r46*), YUC.1 (*Vmn1r49*), YUC.3 (*Vmn1r50*), YUC.5 (*Vmn1r51*) and YUD.4 (*Vmn1r54*). V1R sequences from wild isolates [GenBank: JF782602–JF783819] were compared to the orthologous gene in each strain genome. Five genes were excluded because either no sequence could be generated in some isolates [34] or they did not meet the sequence quality threshold in our dataset. The wild mouse dataset contains two VR sequences per isolate (Park, S.H. and Zhang, J., personal communication), these were paired together and a match was called if at least one was identical to a strain haplotype. When multiple strains had an identical haplotype, the wild-derived strain of the same subspecies was called ahead of the laboratory derived strains or the wild-derived strain from a difference subspecies.

Total counts of synonymous and non-synonymous SNPs across the entire 17 genomes are from the data described in [42] and further personal communication from those authors. The number of non-synonymous SNPs across all 17 genomes total 136,968 in lab-derived strains and 178,126 in wild-derived strains, and the number of synonymous SNPs total 253,181 in lab-derived strains and 361,993 in wild-derived strains. The total number of SNPs in VRs is detailed in Table1. The SNPs per codon densities were calculated from 599,688,770 bp of coding sequence across the entire 17 genomes (Goodstadt, L., personal communication), and 5, 236, 930 bp of VR coding sequence (Table1).

### V2R functional categorisation

V2Rs that respond to conspecific, predator or sympatric cues were identified from [32]. Briefly, Isogai *et al.* exposed *domesticus*-derived mice to olfactory stimuli from each source and then quantified the overlap of *Egr1* positive cells in the VNO (indicating neuronal activation) with *in situ* hybridization of probes against VRs. Specific probes to 29 V2R genes in our parsed dataset co localized with cells responsive to conspecific, predator or sympatric cues. Two V2Rs were excluded because they were responsive to more than one class of stimulus, leaving 11 conspecific, 12 predator and 4 sympatric responsive V2Rs. The coding variation in these genes was compared within 13 *domesticus*-derived strains and the non-*domesticus* strains: CAST/EiJ, PWK/PhJ and SPRET/EiJ.

### Statistical analysis

Two-way ANOVA with repeated measures for strain followed by Bonferroni *post hoc* tests, or two-tailed t-tests, were applied as appropriate using GraphPad Prism 5. Statistical significance was considered when P < 0.05. PCA was performed using non-synonymous SNPs / kb values from 13 lab-derived strains using the publicly available R package for statistical computing, version 2.14.0 [71]. Ten missing values were encountered due to lineage specific deletions/duplications. The average of the two neighbouring genes was used in these cases.

## Abbreviations

CNV: Copy number variation; FPR: Formyl-peptide receptor family; MPS: Massively parallel sequencing; PCA: Principal component analysis; SNP: Single nucleotide polymorphism; V1R: Vomeronasal receptor family 1; V2R: Vomeronasal receptor family 2; VNO: Vomeronasal organ; VR: Vomeronasal receptor; VSN: Vomeronasal sensory neuron.

## Competing interests

The authors declare that they have no competing interests.

## Authors’ contributions

DL conceived the project. EW, KC and DL collected and analysed the V2R data, EW and DL collected and analysed the V1R and FPR data. GSA devised and carried out the principal component and statistical analyses. DL and GSA produced the figures and DL drafted the manuscript. All authors read and approved the final manuscript for publication.

## Supplementary Material

Additional file 1 **Table S1.** Listing the numbers of VRs at each stage of our parsing process, subdivided by receptor class. Click here for file

Additional file 2 **Table S2.** Listing the SNP distribution in VR repertoires, subdivided by receptor class. Click here for file

Additional file 3 **Figure S1.** Showing an example of a VR, *Vmn2r100*, deleted in SPRET/EiJ. (A) Exome sequencing of C3HeB/FeJ indicates the genomic location of the *Vmn2r100* gene (black bars) in that strain, by the position of reads mapped to the C57BL/6J reference (blue lines). (B) The mapping of whole genome sequence reads from SPRET/EiJ to the same genomic interval shows a defined gap in read coverage. This is consistent with a genomic deletion in this strain. (C) The mapping of whole genome sequence reads from CAST/EiJ to the same genomic interval shows that reads span the whole region in this strain, and thus suggests the gap in SPRET/EiJ is not due to a read mapping problem. Click here for file

Additional file 4 **Figure S2.** Showing evidence of a duplication in *Vmn2r56* in the CAST/EiJ line. Sequence reads from CAST/EiJ are stacked vertically, mapped to an exon of *Vmn2r56* on chromosome 7 of the C56BL/6J reference sequence (bottom, black text). SNPs are indicated in red, with three sites showing ambiguous calls (asterisks: sites where approximately half the reads has one nucleotide and the other half has a different nucleotide). The nucleotides at these sites co-segregate within reads (blue text and green text), consistent with two distinct sequences in CAST/EiJ mapping to the same location. Click here for file

Additional file 5 Lists all the SNPs identified in V1Rs, subdivided by gene and strain. Click here for file

Additional file 6 Lists all the SNPs identified in V2Rs, subdivided by gene and strain. Click here for file

Additional file 7 Lists all the SNPs identified in FPRs, subdivided by gene and strain. Click here for file
