# Supplementary material for: Genomic variation in the vomeronasal receptor gene repertoires of inbred mice
Source: BMC Genomics. 2012 Aug 21;13:415. doi: 10.1186/1471-2164-13-415 (PMC3460788; doi:10.1186/1471-2164-13-415)
Supplement: Additional file 1 — Table S1. Listing the numbers of VRs at each stage of our parsing process, subdivided by receptor class. [file 1471-2164-13-415-S1.doc]

## Supplementary Table 1. VR content and their sequence variation by class.

| **VRs:** | **V1Rs** | **V2Rs** | **FPRs** | **Total** |
| --- | --- | --- | --- | --- |
| Searched in NCBIM37 | 239 | 121 | 7 | 367 |
| Identified in NCBIM37 | 239 | 120 | 7 | 366 |
| With data collected over 17 genomes | 4,063 | 2,040 | 119 | 6,222 |
| Lacking full-length coverage | 1705 | 558 | 17 | 2280 |
| Lacking full-length coverage (%) | 41.9% | 27.3% | 14.3% | 36.7% |
| With >50% ambiguous calls | 75 | 135 | 7 | 217 |
| With >50% ambiguous calls (%) | 1.8% | 6.6% | 5.9% | 3.5% |
| Discarded for >3 strains | 303 | 46 | 10 | 359 |
| Discarded for >3 strains (%) | 7.5% | 2.2% | 8.4% | 5.8% |
| Remaining in parsed dataset | 1,980 | 1,301 | 85 | 3,366 |
| Remaining in parsed dataset (%) | 48.7% | 63.7% | 71.4% | 54.1% |
