# Supplementary material for: Genomic variation in the vomeronasal receptor gene repertoires of inbred mice
Source: BMC Genomics. 2012 Aug 21;13:415. doi: 10.1186/1471-2164-13-415 (PMC3460788; doi:10.1186/1471-2164-13-415)
Supplement: Additional file 2 — Table S2. Listing the SNP distribution in VR repertoires, subdivided by receptor class. [file 1471-2164-13-415-S2.doc]

## Supplementary Table 2. SNP distribution in VR repertoires by class.

| **SNPs:** | **V1Rs** | **V2Rs** | **FPRs** | **Total** |
| --- | --- | --- | --- | --- |
| Total after parsing | 3911 | 7012 | 284 | 11207 |
| Non-synonymous after parsing | 2246 | 4010 | 138 | 6394 |
| Non-synonymous after parsing (%) | 57.4% | 57.2% | 48.6% | 57.1% |
| Synonymous after parsing | 1665 | 3002 | 146 | 4813 |
| Synonymous after parsing (%) | 42.6% | 42.8% | 51.4% | 42.9% |
| Per Kb of coding sequence | 2.361 | 2.406 | 3.490 | 2.407 |
| Per Kb (in laboratory strains) | 1.083 | 1.194 | 2.392 | 1.159 |
| Per Kb (in wild-derived strains) | 6.195 | 6.043 | 6.784 | 6.151 |
| Non-synonymous per Kb (lab strains) | 0.635 | 0.681 | 1.147 | 0.666 |
| Non-synonymous per Kb (wild-derived) | 3.516 | 3.452 | 3.343 | 3.487 |
