# Supplementary figures and images for: Genomic variation in the vomeronasal receptor gene repertoires of inbred mice
Source: BMC Genomics. 2012 Aug 21;13:415. doi: 10.1186/1471-2164-13-415 (PMC3460788; doi:10.1186/1471-2164-13-415)

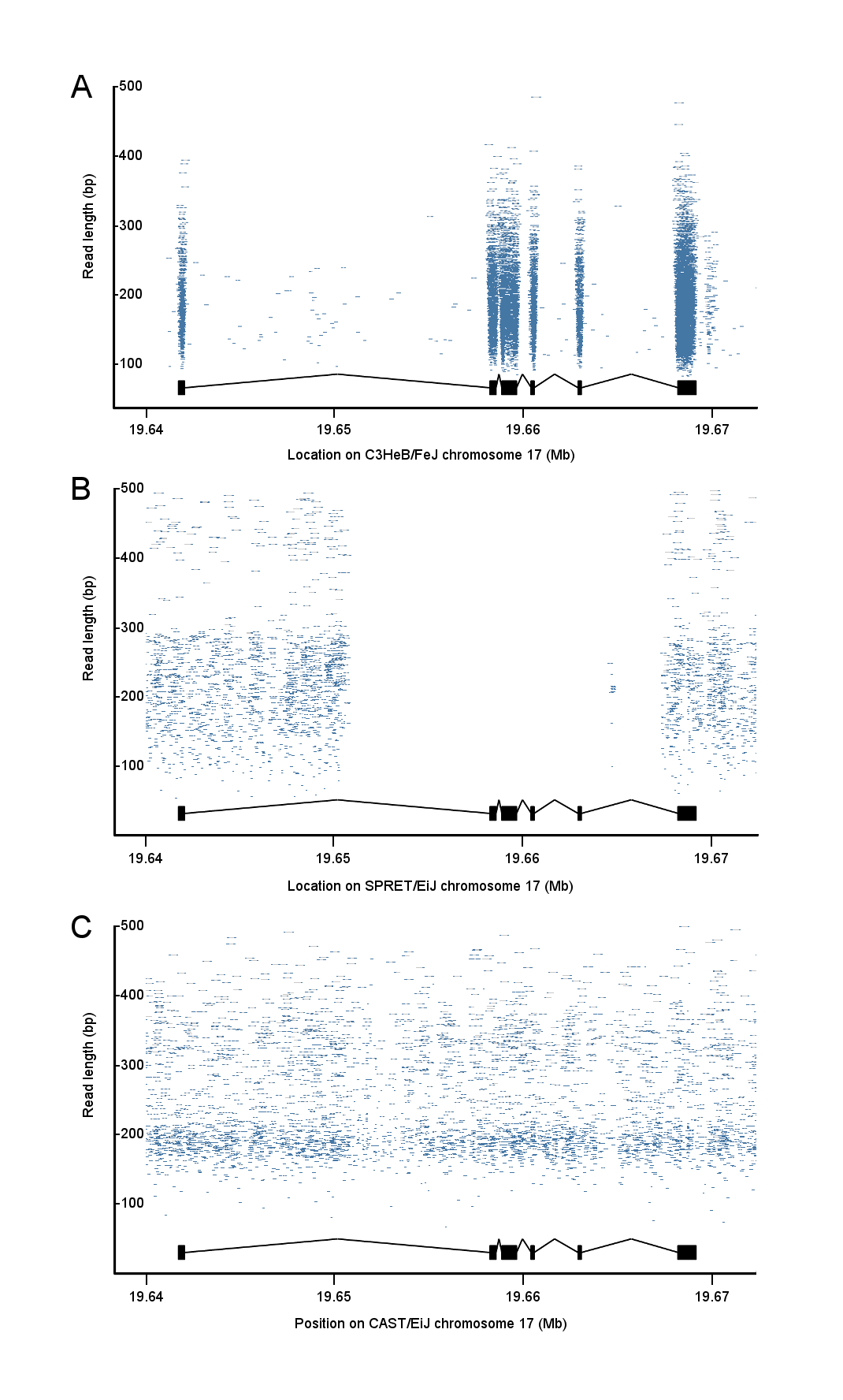

Supplement: Additional file 3 — Figure S1. Showing an example of a VR, Vmn2r100, deleted in SPRET/EiJ. (A) Exome sequencing of C3HeB/FeJ indicates the genomic location of the Vmn2r100 gene (black bars) in that strain, by the position of reads mapped to the C57BL/6J reference (blue lines). (B) The mapping of whole genome sequence reads from SPRET/EiJ to the same genomic interval shows a defined gap in read coverage. This is consistent with a genomic deletion in this strain. (C) The mapping of whole genome sequence reads from CAST/EiJ to the same genomic interval shows that reads span the whole region in this strain, and thus suggests the gap in SPRET/EiJ is not due to a read mapping problem. [file 1471-2164-13-415-S3.tiff]

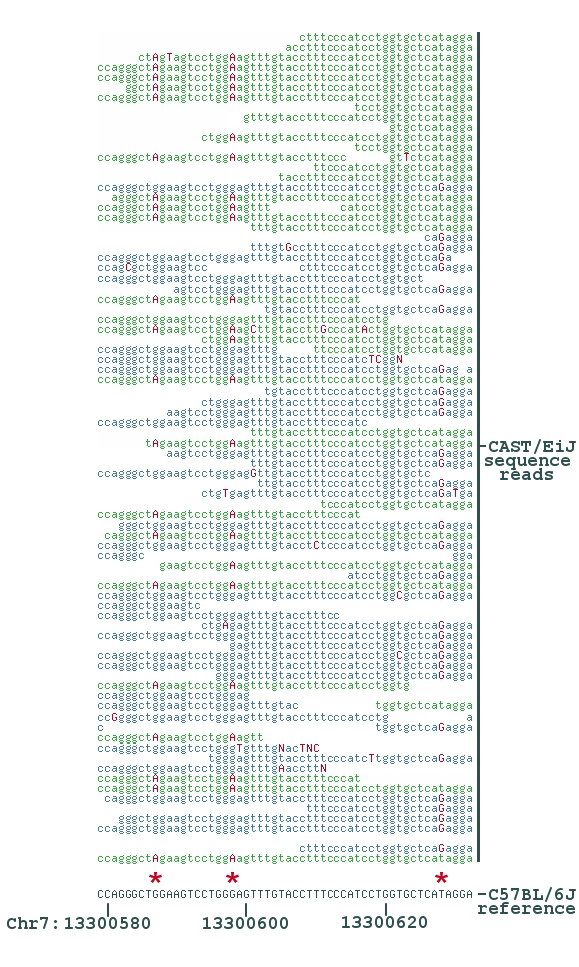

Supplement: Additional file 4 — Figure S2. Showing evidence of a duplication in Vmn2r56 in the CAST/EiJ line. Sequence reads from CAST/EiJ are stacked vertically, mapped to an exon of Vmn2r56 on chromosome 7 of the C56BL/6J reference sequence (bottom, black text). SNPs are indicated in red, with three sites showing ambiguous calls (asterisks: sites where approximately half the reads has one nucleotide and the other half has a different nucleotide). The nucleotides at these sites co-segregate within reads (blue text and green text), consistent with two distinct sequences in CAST/EiJ mapping to the same location. [file 1471-2164-13-415-S4.tiff]
